# Paracrine IFN Response Limits ZIKV Infection in Human Sertoli Cells

**DOI:** 10.3389/fmicb.2021.667146

**Published:** 2021-05-17

**Authors:** Daniel P. Strange, Boonyanudh Jiyarom, Hooman Sadri-Ardekani, Lisa H. Cazares, Tara A. Kenny, Michael D. Ward, Saguna Verma

**Affiliations:** ^1^Department of Tropical Medicine, Medical Microbiology, and Pharmacology, John A. Burns School of Medicine, University of Hawai’i at Mãnoa, Honolulu, HI, United States; ^2^Wake Forest Institute for Regenerative Medicine, Wake Forest School of Medicine, Winston-Salem, NC, United States; ^3^Department of Urology, Wake Forest School of Medicine, Winston-Salem, NC, United States; ^4^Systems and Structural Biology Division, Protein Sciences Branch, U.S. Army Medical Research Institute of Infectious Diseases, Frederick, MD, United States

**Keywords:** ZIKV, testes, Sertoli cells, IFN response, host-ZIKV interaction, ISGs, MX1, IFIT1

## Abstract

Zika virus (ZIKV) is unique among mosquito-borne flaviviruses in its ability to be sexually transmitted. The testes have been implicated as sites of long-term ZIKV replication, and our previous studies have identified Sertoli cells (SC), the nurse cells of the seminiferous epithelium that govern spermatogenesis, as major targets of ZIKV infection. To improve our understanding of the interaction of ZIKV with human SC, we analyzed ZIKV-induced proteome changes in these cells using high-throughput liquid chromatography-tandem mass spectrometry (LC-MS/MS). Our data demonstrated that interferon (IFN) signaling was the most significantly enriched pathway and the antiviral proteins MX1 and IFIT1 were among the top upregulated proteins in SC following ZIKV infection. The dynamic between IFN response and ZIKV infection kinetics in SC remains unclear, therefore we further determined whether MX1 and IFIT1 serve as antiviral effectors against ZIKV. We found that increased levels of MX1 at the later time points of infection coincided with diminished ZIKV infection while the silencing of *MX1* and *IFIT1* enhanced peak ZIKV propagation in SC. Furthermore, although IFN-I exposure was found to significantly hinder ZIKV replication in SC, IFN response was attenuated in these cells as compared to other cell types. The data in this study highlight IFN-I as a driver of the antiviral state that limits ZIKV infection in SC and suggests that MX1 and IFIT1 function as antiviral effectors against ZIKV in SC. Collectively, this study provides important biological insights into the response of SC to ZIKV infection and the ability of the virus to persist in the testes.

## Introduction

Zika virus (ZIKV) is the only mosquito-borne flavivirus known to be sexually transmitted and capable of establishing persistence in the male reproductive tract (Turmel et al., 2016; Hastings and Fikrig, 2017; Moreira et al., 2017), and thus poses new challenges for controlling ZIKV outbreaks and for developing live-attenuated vaccines. Seminal shedding of ZIKV has been reported to occur for more than a year following symptoms resolution and has been associated with alterations in testis-derived hormone levels and sperm parameters (Mansuy et al., 2016; Joguet et al., 2017; Paz-Bailey et al., 2017). These data collectively implicate the testes as probable sites of prolonged ZIKV replication and suggest that persistence may have short-term effects on male reproductive health. Consistent with these findings in humans, ZIKV has been detected in the testes of non-human primates and immunocompromised mice following subcutaneous inoculation, causing extensive testicular damage in the latter (Govero et al., 2016; Ma et al., 2016; Osuna et al., 2016; Hirsch et al., 2017). Interestingly, although ZIKV is persistently shed in semen (Joguet et al., 2017; Paz-Bailey et al., 2017; Bujan et al., 2020), the virus does not cause testicular pain or inflammation in humans and is ultimately cleared without apparent long-term complications. These observations suggest that the testes, which are immune-privileged organs (Chen et al., 2016), can mount local antiviral defenses that eventually resolve the infection without the help of adaptive immune cells. However, the specific mechanisms that facilitate the eventual resolution of ZIKV from the testes remains elusive.

We and others have recently demonstrated that human Sertoli cells (SC), the nurse cells of the seminiferous epithelium that form the so-called blood-testis barrier and govern spermatogenesis (Kaur et al., 2014; Chen et al., 2016), are highly permissive to ZIKV infection (Siemann et al., 2017; Kumar et al., 2018; Strange et al., 2018a). Furthermore, despite the induction of various interferon (IFN)-stimulated genes (ISGs), SC have been shown to support high levels of ZIKV replication in multiple studies (Siemann et al., 2017; Kumar et al., 2018; Strange et al., 2018a). In contrast, other testis cell types, such as peritubular myoid cells (PMC) and spermatogonia stem cells (SSC), were shown to be less permissive to ZIKV infection, while Leydig cells (LC)—the main resident cell type of the testis interstitium responsible for testosterone production—were found to be resistant to the virus (Strange et al., 2019). Our more recent work also indicated that the TAM (Tyro3, Axl, Mer) receptor tyrosine kinase Axl negatively regulates the antiviral state in SC and that inhibition of Axl kinase activity in SC increased the expression of the ISGs *MX1* and *IFIT1* and decreased ZIKV replication, suggesting that these ISGs may antagonize ZIKV propagation (Strange et al., 2019). Together, these studies and observations provide strong evidence that SC are a major cell type of ZIKV propagation and antiviral defenses in the testes.

Our previously reported transcriptomics study provided insights into the potential antiviral defense mechanisms generated in SC against ZIKV (Strange et al., 2018a), including the upregulation of various ISGs and the predicted activation of IFN and PRR signaling pathways. However, it is also well established that ZIKV possesses the ability to antagonize PRR-mediated induction of type I IFNs (e.g., IFN-α/β) and type III IFNs (IFN-λ1–4) and their downstream signaling through targeted degradation of STAT2 (Nelson et al., 2019). Although type I and III IFNs (IFN-I/III) signal through distinct cognate receptors, their pathways converge onto a common transcription factor complex, referred to as ISGF3, that encompasses STAT1, STAT2, and IRF9 (Mesev et al., 2019). Upon activation through IFN-I/III signal transduction, ISGF3 is translocated to the nucleus, where it stimulates the upregulation of various ISGs, including *MX1*, *IFIT1*, and *ISG15* (Grandvaux et al., 2002), which have all been shown to inhibit ZIKV replication in different cell types (Chen et al., 2017; Singh et al., 2019; Wichit et al., 2019). Thus, delineating the dynamic between IFN response and ZIKV replication in SC is vital for understanding the progression of ZIKV infection in the testes.

To gain further insights into how SC respond and exert control over ZIKV in the testes, we first utilized liquid chromatography-tandem mass spectrometry (LC-MS/MS) proteome analysis to discern the top antiviral proteins upregulated in SC at the early and later time points of ZIKV infection. We next carefully examined ZIKV replication kinetics and IFN response in SC to better define the temporal interplay between these two processes. Subsequently, we performed *MX1* and *IFIT1* knockdown experiments to determine whether these ISGs in particular serve as antiviral effectors against ZIKV in SC. Finally, we compared the ability of SC to respond to IFN-I to that of other human cell types in order to investigate whether IFN-I response in SC is equally as robust as in other permissive cell types.

## Materials and Methods

### Cell Culture and Virus Infection

Primary human SC were obtained from iXCells Biotechnologies (catalog number 10HU-149) and were cultured using a 1:1 Dulbecco’s modified Eagle’s medium (DMEM) to F-12 medium supplemented with HEPES, L-glutamine, 100 U/mL penicillin-streptomycin, and 5% fetal bovine serum (FBS) as described previously (Siemann et al., 2017). A549 cells were cultured using DMEM supplemented with 1 mM sodium pyruvate, 2 mM L-glutamine, 100 U/mL penicillin-streptomycin, 1X non-essential amino acids, 10 mM HEPES, and 10% fetal bovine serum (FBS). HBMVEC obtained from Cell Systems Corporation (catalog number CSC-2M1) were cultured using Endothelial Growth Medium (Angio-Proteomie, catalog number cAP-02). ZIKV strain PRVABC59 (Human/2015/Puerto Rico), acquired from American Type Culture Collection (ATCC), was propagated once in Vero E6 cells for virus stock preparation. ZIKV infection experiments were conducted by exposing cells to the virus at a multiplicity of infection (MOI) of 1 or 3 for 1 h at 37°C and 5% CO_2_. The cells were subsequently washed with phosphate buffered solution (PBS) prior to the addition of fresh media. ZIKV progeny in cell supernatant and intracellular ZIKV RNA was quantified by plaque assay and RT-qPCR, respectively, as reported previously (Verma et al., 2009; Adams Waldorf et al., 2016).

### Proteomics

Total protein was extracted from three biological replicates for each mock and ZIKV-infected SC grown in 6-well tissue culture plates using mammalian protein extraction reagent (M-PER; Thermo Fisher Scientific, 78503) supplemented with protease inhibitor (Thermo Fisher Scientific, A32963). The protein extracts were centrifuged at 14,000 × g at 4°C for 20 min and the supernatant was then removed and transferred to fresh collection tubes and stored at −80°C until processed for tandem mass tag (TMT) 6-Plex labeling using the iFASP method (McDowell et al., 2013; Ward et al., 2019). TMT sample preparation and subsequent LC-MS/MS analysis were performed as described elsewhere (Ward et al., 2019). Acquired MS/MS protein searches were conducted using ProteomeDiscoverer 2.1 Sequest HT (Thermo Fisher Scientific) and the human (taxID 9606) subset of the SwissProt database. Peptide-level false discovery rate (FDR) was set to 0.1% using Posterior Error Probability validation. Only proteins with at least 2 Peptide Spectral Matches (PSM) were considered. Total peptide amount was used for normalization. Mass tolerances were 10 ppm for the MS1 scan and 0.6 Da for all MS/MS scans. Only filtered quantitation results with high-confidence unambiguous PSMs with MS2 isolation interference values of ≤30% were used. The mass spectrometry proteomics data have been deposited to the ProteomeXchange Consortium via the PRIDE partner repository with the dataset identifier PXD025133.

### Pathway Analysis

For pathway enrichment, the lists of differentially regulated proteins (DRPs) were imported into the g:Profiler web server via the g:GOSt functional profiling query list option^1^ under organism Homo sapiens. Statistical domain scope was set to only include annotated genes/proteins and the significance threshold was set to g:SCS with an adjusted *p*-value of < 0.05. Only pathways under the Reactome reference annotation were used.

### Immunofluorescence Assay

SC were grown on 12 mm glass coverslips in 24-well tissue culture plates, exposed to ZIKV at MOI 3, and then fixed at different time points post-infection with 4% paraformaldehyde. The cells were permeabilized with 0.1% triton X-100 in PBS for 10 min, blocked with 5% bovine serum albumin (BSA) in PBS for 30 min, and then incubated with primary antibodies in a 1% BSA in PBS solution for 1 hr. The cells were then washed thrice with PBS, followed by incubation with fluorophore-conjugated secondary antibodies in a 1% BSA in PBS solution for 1 h, washed thrice with PBS, and examined using a Zeiss Axiovert 200 microscope (Verma et al., 2009). ZIKV infection was evaluated using a ZIKV-E mouse monoclonal antibody (1:250 dilution) produced by the Kapi’olani Community College Monoclonal Antibody Service Facility and Training Center (Honolulu, HI) using hybridoma technology and a recombinant ZIKV-E protein produced and purified as previously reported (To et al., 2018). The secondary antibody used for ZIKV-E staining was the Alexa Fluor 488-conjugated sheep anti-mouse (Invitrogen; 1:500 dilution). MX1 protein levels were evaluated using MXA rabbit polyclonal antibody (sc-50509, Santa Cruz; 1:500 dilution). The secondary antibody used for MX1 staining was the Alexa Fluor 594-conjugated goat anti-rabbit (Invitrogen; 1:500 dilution). Mean fluorescence intensity (MFI) of MX1 immunostaining was measured using ImageJ software^2^. MFI fold-change was normalized to mock control. The percentage of ZIKV-positive cells was determined by dividing the number of cells positive for ZIKV-E by the number of total nuclei (stained with DAPI) per field.

### Western Blot and ELISA

Western blot analysis was conducted on total protein extracted from mock and ZIKV-infected SC grown in 6-well tissue culture plates as described previously (Kumar et al., 2013). β-actin and IRF3 were used as loading controls and were detected using β-actin mouse monoclonal antibody (8H10D10, Cell Signaling Technology; 1:1,000 dilution) and IRF3 rabbit polyclonal antibody (11904, Cell Signaling Technology; 1:1,000 dilution). MX1 was detected using MX1 rabbit polyclonal antibody (sc-50509, Santa Cruz; 1:1,000 dilution). Secondary antibodies (1:10,000 dilution) were conjugated with IRDye 800 and IRDye 680 (Li-Cor Biosciences), and blots were scanned using an Odyssey infrared imager. For IFN-β ELISA, secreted IFN-β in SC supernatant was quantified using an enzyme-linked immunosorbent assay (ELISA) kit (R&D Systems DY814-05) according to the manufacturer protocol.

### Gene Silencing and RT-qPCR

Gene silencing of *MX1* and *IFIT1* was performed using Silencer Select siRNAs (Thermo Fisher Scientific) s9101 (MX1) and s7150 (IFIT1), respectively. The Silencer Select negative control No. 1 siRNA (Thermo Fisher Scientific) was used as the control. Cells were transfected with the siRNA using Lipofectamine transfection reagent (Invitrogen) according to the manufacturer’s protocol 24 h prior to ZIKV exposure at MOI 1. Following a 1-h exposure to the virus, the cells were washed with PBS and then 50% of fresh media and 50% of media containing the transfection reagent was returned to the wells. For gene expression analyses, total RNA was extracted from mock and ZIKV-infected cell lysates using RNeasy Mini Kit (Qiagen) and then synthesized into cDNA using qScript cDNA synthesis kit (Quantabio). Changes in mRNA transcripts of ISGs were measured by RT-qPCR, as described previously (Verma et al., 2009), using primers outlined in Table 1. The housekeeping gene *GAPDH* was used to normalize fold-change values with respective mock as the reference control.

**TABLE 1:** Primers used for RT-qPCR.

**Gene accession no.**	**Primer direction**	**Primer sequence (5′–3′)**
*MX1*	F	AGTATGGTGTCGACATACCGGA
NM_001282920.1	R	GAGTCTGGTAAACAGCCGAAT
*IFIT1*	F	TCAGGTCAAGGATAGTCT
NM_001548.5	R	TGTATTTGGTGTCTAGGAAT
*ISG15*	F	AATGCGACGAACCTCTGA
NM_005101	R	GCTCACTTGCTGCTTCAG
*IFNB1*	F	CTCTCCTGTTGTGCTTCTCC
NM_002176.4	R	GTCAAAGTTCATCCTGTCCTTG
*IFNL3*	F	CTGACGCTGAAGGTTCTG
NM_001346937	R	GCTGGGAGAGGATATGGT
*GAPDH*	F	AGTCAGCCGCATCTTCTTTTGC
NM_002046.7	R	CAATACGACCAAATCCGTTGACT

### Exogenous IFN-β Treatment

For ZIKV inhibition assay (Figure 2), SC cultured in 48-well plates were treated with 5 pg/mL (1 IU/mL) of recombinant human IFN-β (rhIFN-β; R&D Systems, 8499-IF) for 24 h prior to ZIKV infection at MOI 1. The cells were continually exposed to rhIFN-β during and after ZIKV infection and were replenished with rhIFN-β at 24 h post-infection. SC lysates were then collected at 48 h post-infection to determine differences in ZIKV copy number and ISG expression in comparison to untreated controls via RT-qPCR analysis. For IFN-I response assay (Figure 4), SC, A549 cells, and HBMVEC cultured in 24-well plates at equal density were treated with 30 pg/mL (6 IU/mL) of rhIFN-β and then lysates were collected 24 h later to determine differences in ISG expression in comparison to untreated controls via RT-qPCR analysis.

### Statistical Analysis

Statistical analyses were performed using GraphPad Prism (GraphPad Software, San Diego, CA). For ZIKV titers, mean fluorescence intensity (MFI), ELISA, and RT-qPCR data, statistical differences were determined by an unpaired Student’s *t*-test; a *p*-value of < 0.05 was considered significant, and error bars denote mean ± standard deviation (SD) of data from ≥3 independent experiments. For protein abundance data, a direct comparison was made to time-matched mock samples to determine significance using one-way ANOVA. A *p*-value adjustment was then performed for multiple comparisons using the two-stage linear step-up procedure of Benjamini, Krieger, and Yekutieli and a false discovery rate (FDR) of 5%. Only abundance ratios of >1.6 or <0.6 with an adjusted *p* < 0.05 were considered significant.

## Results

### MX1 and IFIT1 Are the Top Upregulated Proteins in Sertoli Cells During ZIKV Infection

**FIGURE 1:**
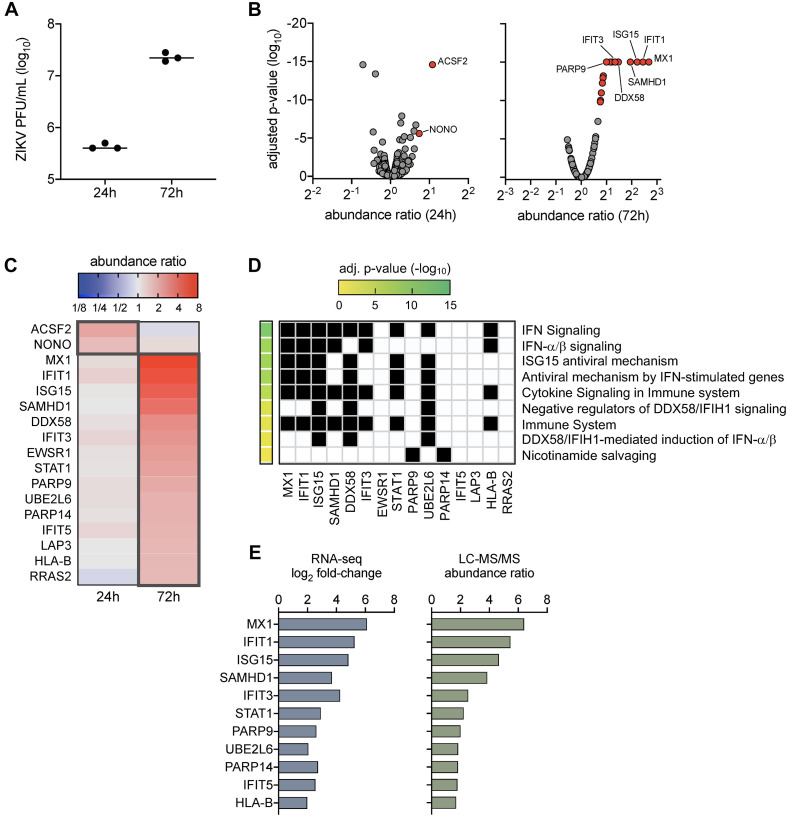
Proteome profiles of Sertoli cells following ZIKV infection. **(A)** ZIKV titers in SC were quantified by plaque assay at 24 and 72 h post-infection (1 h exposure at MOI 3). **(B–D)** Proteome analysis was conducted on mock (*n* = 3) and infected (*n* = 3) SC at 24 and 72 h post-infection using the LC-MS/MS platform. Acquired MS/MS protein searches were performed using ProteomeDiscoverer 2.1 Sequest HT (Thermo Fisher Scientific) and human (taxID 9606) subset of the SwissProt database. Protein abundance ratios (ZIKV/mock) were determined by direct comparison of time-matched samples (*n* = 3 for each mock and infected at each time point). Significance determined using one-way ANOVA and *p*-values adjusted for multiple comparisons using two-stage linear step-up procedure of Benjamini, Krieger, and Yekutieli, false discovery rate (FDR) of 5%. **(B)** Volcano scatter plot of abundance ratio versus adjusted *p*-value of identified proteins at 24 and 48 h post-infection. Only protein abundance ratios of > 1.6 or < 0.6 with an adjusted *p* < 0.05 were considered significant. Gray data points represent abundance ratios that were insignificant, red data points represent upregulated proteins, and blue data points represent downregulated proteins. **(C)** Heatmap comparison of the differentially regulated proteins (DRPs; proteins with significant abundance ratios) at 24 and 72 h post-infection. Dark gray outline denotes DRPs for each time point. **(D)** Pathway enrichment matrices of 72 h DRPs were determined using Reactome annotations (https://reactome.org) within the g:Profiler web server, g:GOSt functional profiler option (https://biit.cs.ut.ee/gprofiler/gost). Significance threshold was set to g:SCS with adjusted *p*-value of < 0.05. **(E)** Comparison profile between DRPs and their encoding differential expressed genes (DEGs) identified using RNA-Seq analysis in our previous study in ZIKV-infected SC at 72 h post-infection.

Our prior work demonstrated that ZIKV titers peaked in SC by 72 h post-infection (Siemann et al., 2017; Strange et al., 2018a), which also corresponded with the strong induction of genes involved in innate antiviral defense pathways (Strange et al., 2018a). To further evaluate the impact of ZIKV infection in SC at the protein level, here we conducted LC-MS/MS proteomics on SC at 24 and 72 h post-infection at a multiplicity of infection (MOI) of 3. Plaque assay was also performed to confirm infection and indicated that ZIKV titers increased in SC by more than 1.5 log_10_ from 24 to 72 h post-infection (Figure 1A). LC-MS/MS proteome analysis detected a total of 1,281 cellular proteins with 99% confidence at the peptide and protein levels. The statistical cutoff for differentially regulated proteins (DRPs) in infected cells was set to an abundance ratio of more than 1.6 and less than 0.6 with an adjusted *p*-value of less than 0.05 in comparison to mock (Figure 1B). Using this criterion, 2 and 15 DRPs were identified in SC at 24 and 72 h post-infection, respectively (Figure 1C and Supplementary Tables 1, 2).

To determine the function of identified DRPs, we next conducted biological pathway enrichment analysis using the Reactome database within the g:Profiler webserver. The two DRPs identified at 24 h post-infection, ACSF2 and NONO, did not yield pathway enrichment results. However, analysis of DRPs enriched at 72 h indicated that these proteins were predominantly involved in innate antiviral defense pathways, including IFN signaling, ISG15 antiviral mechanism, antiviral mechanism by ISGs, and DDX58/IFIH1 signaling and regulation (Figure 1D). This analysis was largely consistent with pathways enriched by our previously reported RNA-seq analysis at the same time point of infection (Strange et al., 2018a). Thus, to help provide validation to these results, we next compared the 72 h DRPs with the 72 h differentially expressed genes (DEGs) from our RNA-seq analysis (Strange et al., 2018a) and found an overlap of 73% (11/15 DRPs) (Figure 1E). These included various ISG-encoded proteins reported to have antiviral potential against ZIKV such as MX1, IFIT1, and ISG15 (Chen et al., 2017; Singh et al., 2019; Wichit et al., 2019), as well as other ISG-encoded proteins such as SAMHD1, IFIT3, STAT1, UBE2L2, IFIT5, and HLA-B. Collectively, these data indicate that proteins involved in innate antiviral defense are the most prominent dysregulated proteins in SC during peak ZIKV infection, with MX1 and IFIT1 as the top upregulated proteins detected.

### IFN Response Limits ZIKV Infection in Sertoli Cells

**FIGURE 2:**
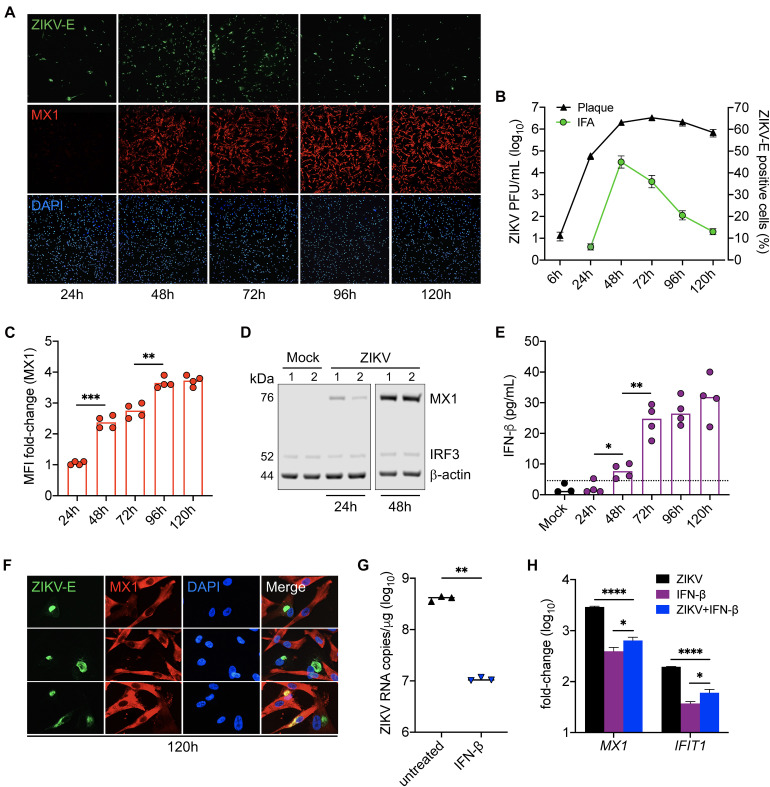
IFN response limits ZIKV infection in Sertoli cells. SC were exposed to ZIKV for 1h (MOI 3) and ZIKV infection and MX1 protein levels were evaluated by **(A)** immunofluorescence assay (IFA) using mouse monoclonal against the ZIKV E protein (green) and rabbit polyclonal against MX1 (red), respectively. Nuclei were stained with DAPI (blue). Images were taken at 100× magnification. **(B)** ZIKV titers in SC supernatant were (black line curve) determined by plaque assay (*n* = 3 for each time point) and the percentage of ZIKV-positive cells (green line curve) was quantified via IFA (*n* = 2 field per coverslip for each time point). **(C)** Changes in MX1 protein levels were quantified by mean fluorescence intensity (MFI) of IFA coverslips (*n* = 4) from each time point using ImageJ software, reported as MFI fold-change compared to mock. **(D)** Western blot analysis of MX1 protein levels at 24 and 48 h post-infection. b-actin and IRF3 were used as loading controls and each lane represents an independent experiment. **(E)** Secreted IFN-β in SC supernatant (*n* = 4 for each time point), determined by human IFN-β ELISA (R&D Systems); dotted line denotes detection limit of assay. **(F)** Zoomed images of IFA to depict co-localization of ZIKV and MX1 at 120h post-infection. **(G,H)** SC were treated with 5 pg/mL (1 IU/mL) of recombinant human IFN-β (rhIFN-β; R&D systems) 24 h prior to and upon ZIKV infection (MOI 1) and were replenished with the rhIFN-β (5 pg/mL) at 24 h post-infection. **(G)** ZIKV genome copies were measured in infected SC with and without IFN-β treatment at 48 h post-infection by RT-qPCR. **(H)** Gene expression of *MX1* and *IFIT1* was evaluated at 48 h post-infection by RT-qPCR in both mock and infected SC with and without IFN-β treatment and reported as fold-change compared to mock untreated. The housekeeping gene *GAPDH* was used to normalize fold-change for all gene expression assays. Significance determined by Student’s *t*-test for all assays, ^∗^*p* < 0.05, ^∗∗^*p* < 0.01, ^∗∗∗^*p* < 0.001, ^****^p < 0.0001.

LC-MS/MS analysis identified MX1 as the top upregulated antiviral protein in SC during peak ZIKV infection. MX1 is an ISG-encoded protein exclusively upregulated by both IFN-I and III signal transduction (Verhelst et al., 2013) and thus serves as a marker of an antiviral state triggered through IFN-I/III pathways. To better elucidate the dynamics between ZIKV infection and the antiviral state in SC, we next sought to evaluate ZIKV infection kinetics and its association with MX1 protein levels in SC over time. To accomplish this, we first exposed SC to ZIKV for 1 h and then measured ZIKV envelope (E) and MX1 protein levels by immunofluorescence assay (IFA) and ZIKV infectious progeny in the supernatant by plaque assay each day for up to 5 days post-infection. We found that the percentage of SC positive for ZIKV-E peaked by 48 h post-infection, whereas plaque assay conducted on the same samples showed that infectious ZIKV progeny peaked in the supernatant by 72 h post-infection (Figures 2A,B). Consistent with our LC-MS/MS data, IF staining showed that MX1 protein was nearly undetectable at 24 h, but then increased significantly by 48 h post-infection (Figures 2A,C). Western blot analysis also showed that MX1 levels increased significantly at 48 h post-infection (Figure 2D). IF data indicated that MX1 protein levels were most elevated between 96h and 120h post-infection (Figures 2A,C) and this coincided with diminishing ZIKV-positive SC (Figures 2A,B). Increasing MX1 levels also corresponded with the detection of secreted IFN-β in the supernatant, as measured by ELISA, which increased steadily up to 72 h and then remained elevated up to 120 h post-infection (Figure 2E). Although a majority of SC expressed MX1 by 120 h post-infection, we found that SC that were positive for ZIKV-E exhibited low levels of MX1 in comparison to the strong signal observed in neighboring uninfected cells (Figure 2F). Collectively, these results indicate that IFN response is strongly triggered in bystander SC following ZIKV infection, and therefore suggests that IFN response in naïve SC may exert control over the spread of ZIKV in the seminiferous epithelium.

Since the upregulation of MX1 was most prominent in naïve SC at the later timepoints of infection (Figure 2A), we next questioned whether IFN-I signaling could be protective against ZIKV in SC if triggered prior to infection. To investigate, SC were pretreated with 5 pg/mL (1 IU/mL) of recombinant human IFN-β for 24 h prior to infection (MOI 1) and ZIKV replication as well as *MX1* and *IFIT1* expression were then evaluated by RT-qPCR at 48 h post-infection. We found that the SC primed with IFN-β exhibited more than a 1.5 log_10_ (>95%) reduction in ZIKV genome copies compared to untreated control (Figure 2G). Furthermore, both IFN-β treatment and ZIKV infection induced the expression of *MX1* and *IFIT1* (Figure 2H). These results confirm that SC are less permissive to ZIKV infection following triggered IFN-I response and suggest that IFN-β is a major driver of the antiviral state in SC following ZIKV infection.

### Silencing of *MX1* and *IFIT1* Enhances Peak ZIKV Propagation in Sertoli Cells

**FIGURE 3:**
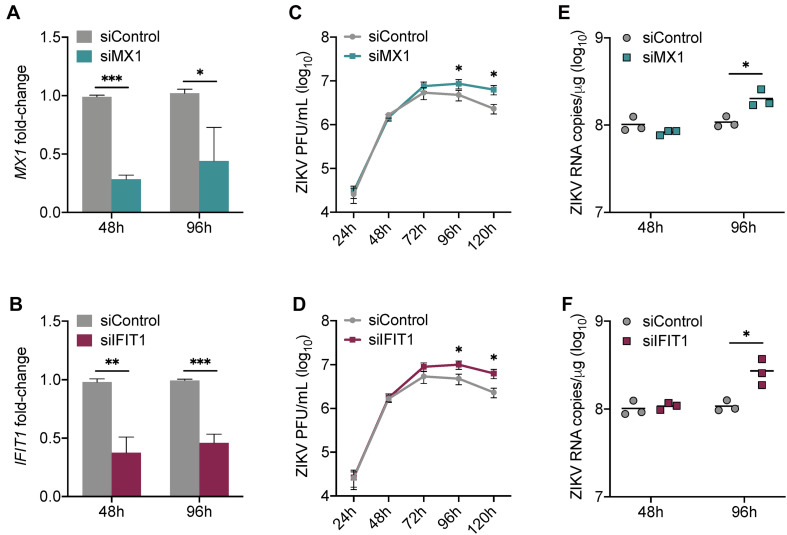
Silencing of *MX1* and *IFIT1* enhances peak ZIKV replication in Sertoli cells. SC were transfected with siRNA for *MX1* (siMX1), *IFIT1* (siIFIT1), or negative control (siControl) 24 h prior to ZIKV infection (MOI 1). **(A)** The silencing of *MX1* (siMX1) and **(B)** IFIT1 (siIFIT1) was evaluated at 48 and 96 h post-infection by measuring *MX1* and *IFIT1* expression, respectively, in mock SC, determined by RT-qPCR and reported as fold-change compared to siControl. **(C,D)** ZIKV infectious progeny measured in infected SC transfected with siControl and siMX1 or with siControl and siIFIT1 by plaque assay. **(E,F)** ZIKV genome copies in infected siControl were compared to **(E)** infected siMX1 and **(F)** infected siIFIT1 by RT-qPCR. The housekeeping gene *GAPDH* was used to normalize fold-change for all gene expression assays. Significance (*n* = at least 3 for each condition at each time point) determined by Student’s *t*-test for all assays, ^∗^*p* < 0.05, ^∗∗^*p* < 0.01, ^∗∗∗^*p* < 0.001.

Our data collectively suggest that MX1 and IFIT1 are potent markers of the antiviral state in SC in response to ZIKV infection. To investigate whether MX1 and IFIT1 actively antagonize ZIKV propagation in SC, we next transfected SC with either *MX1* or *IFIT1* siRNA 24 h prior to infection (MOI 1). This resulted in significant silencing of *MX1* and *IFIT1* at 48 h post-infection (72 h post-transfection) that remained up to 96 h post-infection (Figures 3A,B). However, the silencing of these genes even prior to ZIKV infection did not affect virus replication kinetics until 72 h post-infection and a significant increase in ZIKV infectious progeny was not observed until 96 h and 120 h post-infection as compared to siControl (Figures 3C,D). The impact of siMX1 and siIFIT1 was further evaluated by measuring ZIKV genome copies at 48 and 96 h post-infection and confirmed that *MX1* and *IFIT1* silencing only affected ZIKV RNA replication at the later time point of infection (Figures 3E,F). These data indicate that MX1 and IFIT1 participate in controlling ZIKV propagation in SC, particularly at the later time points of infection.

### IFN Response in Sertoli Cells Is Dampened in Comparison to Other Human Cell Types

**FIGURE 4:**
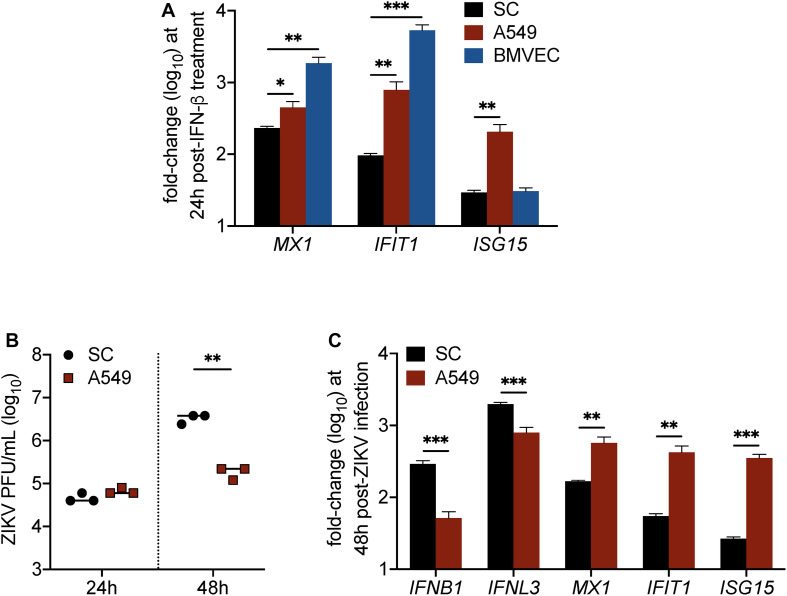
IFN response is less robust in Sertoli cells as compared to brain microvascular endothelial cells and A549 lung epithelial cells. **(A)** Human SC, BMVEC, and A549 cells were treated with 30 pg/mL (6 IU/mL) of recombinant human IFN-β (rhIFN-β; R&D systems), RNA was extracted from cell lysates 24 h later, and gene expression of ISGs (*MX1*, *IFIT1*, and *ISG15*) was measured by RT-qPCR (*n* = 3 for each infected and mock samples). **(B,C)** SC and A549 cells were infected with ZIKV at MOI 1. **(B)** ZIKV progeny was measured in the culture supernatant by plaque assay at 24 and 48 h post-infection. **(C)** Expression of mRNA transcripts for type I IFN (*IFNB1*), type III IFN (*IFNL3*), *MX1*, *IFIT1*, and *ISG15* in ZIKV-infected SC and A549 cells compared to respective mock samples was measured by RT-qPCR at 48 h post-infection (*n* = 3 for each infected and mock samples). The housekeeping gene *GAPDH* was used for normalization for all gene expression analyses. Significance determined by Student’s *t*-test for all assays, ^∗^*p* < 0.05, ^∗∗^*p* < 0.01, ^∗∗∗^*p* < 0.001.

Although data in Figures 2, 3 indicated that IFN response in SC limits ZIKV infection, we also found that IFN response, as demonstrated by MX1 protein levels, was not strongly detected in SC until infection reached its peak (Figure 2). This observation raised the question as to whether SC respond to IFN as robustly as other human cell types. To investigate this, we next compared the induction of ISGs in SC to that of A549 human lung epithelial cells and human brain microvascular endothelial cells (HBMVEC), which are two cell types also shown to be permissive to ZIKV infection (Siemann et al., 2017; Kumar et al., 2018), following stimulation with IFN-β. Gene expression of the ISGs *MX1*, *IFIT1*, and *ISG15* was measured in all of these cell types at 24 h following treatment with 30 pg/mL (6 IU/mL) of recombinant human IFN-β. We found that *MX1* and *IFIT1* were most induced in BMVEC following IFN treatment (Figure 4A), whereas A549 cells displayed significantly higher induction of all three ISGs in comparison to SC (Figure 4A). These results suggest that SC exhibit dampened IFN-I response as compared to A549 cells and BMVEC.

Considering that these ISGs have been shown to inhibit ZIKV infection individually, we hypothesized that A549 cells, which exhibit higher induction of all three ISGs compared to SC, would be more efficient in restricting ZIKV infection as compared to SC. To investigate, we infected both cell types, seeded at equal density, with ZIKV (MOI 1) and subsequently measured ZIKV propagation by plaque assay at 24 and 48 h post-infection. We observed no significant difference at 24 h; however, by 48 h post-infection, ZIKV titers were significantly lower in A549 cells as compared to SC (Figure 4B). Corresponding to the ZIKV plaque titers at 48 h, we also found that *IFNB1* and *IFNL3* transcript levels were significantly higher in SC as compared to A549 cells (Figure 4C). Conversely, however, but consistent with our IFN-β treatment assay, *MX1*, *IFIT1*, and *ISG15* transcript levels were significantly higher in A549 cells as compared to SC (Figure 4C), indicating that even though ZIKV infection triggered higher IFN induction, it did not translate into stronger downstream IFN signal transduction. Together, these results confirm that IFN response is less robust in SC as compared to A549 cells and suggest that the disparity in these cell types to support ZIKV replication may be tied to their ability to timely induce ISGs following infection.

## Discussion

Recent studies have made substantial progress in our understanding of ZIKV infection in the testes. Our previous work has employed different 2D and 3D human *in vitro* model systems to show that ZIKV infects multiple testis cell types (Strange et al., 2019) and impairs critical testicular processes (Strange et al., 2018b). Previously reported transcriptomics analyses (Kumar et al., 2018; Strange et al., 2018a) demonstrated that SC mounted robust antiviral defense mechanisms in response to ZIKV infection through induction of various ISGs, and also indicated that ZIKV may disrupt canonical pathways involved in germ cell trafficking. In this study, we first took a complementary approach, utilizing LC-MS/MS proteomics to further identify ISG-encoded proteins induced by ZIKV in SC. This was followed by a series of experiments to discern the dynamic between IFN response and ZIKV infection in SC over time. Our data collectively highlights that (i) ZIKV infection in SC primarily impacts pathways involved in innate antiviral defense and IFN signaling, (ii) the top upregulated proteins during ZIKV infection in SC are encoded by the ISGs *MX1* and *IFIT1*, (iii) both MX1 and IFIT1 serve as antiviral effectors against ZIKV in SC, and (iv) the IFN-I response in SC specifically restricts ZIKV infection but is dampened as compared to A549 cells and BMVEC.

The proteins upregulated by ZIKV infection in SC at 72 h post-infection were predominantly antiviral in nature (Figure 1) and the majority of them overlapped with the expression profile of their respective encoding ISGs previously reported (Strange et al., 2018a). These included MX1, IFIT1, ISG15, SAMHD1, IFIT3, and STAT1 (Figure 1), all of which have been shown to be similarly upregulated by ZIKV infection in other human cell types, including fibroblasts (Wichit et al., 2019) and pluripotent stem cell (iPSC)-derived neural progenitor cells (NPC) (Scaturro et al., 2018), as also demonstrated by LC-MS/MS analysis. Surprisingly, however, LC-MS/MS studies involving ZIKV infection of human fetal NPC (Jiang et al., 2018) as well as human mesenchymal stem cells (Beys-da-Silva et al., 2019) did not report upregulation of these ISG-encoded proteins in their data. This may suggest that the antiviral response to ZIKV is cell type-specific, but considering that ZIKV NS proteins antagonize the IFN-I/III pathways in infected cells, the disparity observed across cell types may also be due to differences in the host-viral interaction in different cell types. However, it is also important to note that, in contrast to SC, ZIKV-infected NPC cultures undergo different degrees of cell death and experience impairments in cellular growth depending on their state of differentiation (Wells et al., 2016; Wen et al., 2017), and thus it is plausible that the antiviral responses in NPC may be similarly affected in this regard. In contrast to our LC-MS/MS proteomics analysis, Rashid and colleagues recently employed the SOMAscan aptamer-based multiplexed proteomics approach, that targets a predefined set of proteins, to characterize proteins dysregulated by ZIKV in SC (Rashid et al., 2020). However, although their study reported altered levels related to cell growth, death, and survival in infected SC (Rashid et al., 2020), they could not detect changes in ISG-encoded proteins since they were not included in the predefined set of protein targets of the SOMAscan aptamer-based platform.

Type I and III IFN production and subsequent induction of ISGs are the primary defense mechanisms elicited by most cells against viral intruders (Mesev et al., 2019). However, it is well-established that the NS5 protein of ZIKV antagonizes IFN-I/III signal transduction through targeted degradation of STAT2 (Grant et al., 2016; Kumar et al., 2016; Bowen et al., 2017), a critical component of the IFN-I/III transducer complex. Despite this, IFN signaling, as highlighted by the production of ISG-encoded proteins such as MX1, IFIT1, ISG15, and STAT1 (Grandvaux et al., 2002), was the most significantly enriched pathway in ZIKV-infected SC (Figure 1). Subsequent experiments also revealed that the corresponding increase in MX1 and IFN-β levels was inversely related to ZIKV propagation in SC, and that the priming of IFN-I response in SC prior to infection significantly hindered ZIKV replication (Figure 2). From these data, it is evident that the IFN-I response in bystander cells is important for limiting the spread of ZIKV in SC. However, interestingly, although nearly all infected and naïve SC appeared to express MX1 protein by 120 h post-infection, more than 10% of cells were still producing virus (with ∼10^6^ PFU/mL of infectious progeny detected in the supernatant) suggesting that a limited amount of infected SC retain the ability to replicate virus at high levels. Moreover, despite priming SC with IFN-β prior to infection, we still detected nearly 10^7^ genomic copies of ZIKV at 48 h post-infection (Figure 2), further indicating that ZIKV can overcome the antiviral state in some cells to replicate, which is important in the context of testicular persistence.

Some of the ISG-encoded proteins detected in our analysis, specifically MX1, IFIT1, and ISG15, have been shown to individually exert antiviral activity against ZIKV in different human cell types, including trophoblasts (Chen et al., 2017), fibroblasts (Wichit et al., 2019), and corneal epithelial cells (Singh et al., 2019). In all of these studies, silencing of these genes demonstrated an increase in ZIKV propagation by 48 h post-infection (Chen et al., 2017; Singh et al., 2019; Wichit et al., 2019). MX1 is known to be exclusively induced by IFN-I/III signal transduction (Grandvaux et al., 2002; Holzinger et al., 2007), whereas IFIT1 can be induced in an IFN-dependent and -independent manner (Grandvaux et al., 2002). While MX1 and IFIT1 were the top upregulated proteins in SC following ZIKV infection (Figure 1), a major highlight of this study was that these proteins were also shown to restrict ZIKV replication in SC (Figure 3). An intriguing distinction, however, between our SC data and studies of other human cell types (Chen et al., 2017; Wichit et al., 2019) is that the effect of silencing these genes on ZIKV infection was not significantly apparent until later in infection (Figure 3), 96 h versus 48 h, respectively. The reason for this is unclear, but we speculate that the inherent immunosuppressive nature of SC may be involved, as these cells are known to exhibit restrained innate immune responses to pathogens as well as to exogenous signals due to their important role in governing spermatogenesis (Kaur et al., 2014; Chen et al., 2016). Nonetheless, our *MX1* and *IFIT1* silencing data reinforces the notion that paracrine IFN response in SC, when at its peak (96—120 h), provides resistance for neighboring cells not yet infected.

In general, ZIKV is shown to strongly trigger IFN response in many human cell types, including skin fibroblasts, Hofbauer cells, dendritic cells, macrophages, and A549 cells, by as early as 24 h post-infection (Hamel et al., 2015; Quicke et al., 2016; Bowen et al., 2017; Carlin et al., 2018; Zimmerman et al., 2018; Esser-Nobis et al., 2019). However, it is clear from our data here that the ZIKV-induced antiviral state in SC does not become evident until 48 h post-infection, when ZIKV titers begin to peak and more than 40% of the cells are producing virus (Figure 2). We speculate that this apparent delay, in addition to the ability for ZIKV to antagonize IFN-I/III response in infected cells (Grant et al., 2016; Kumar et al., 2016; Bowen et al., 2017), may be in part due to restrained innate immune responses of SC mentioned above, as evidenced by our data demonstrating dampened IFN-I response in SC as compared to A549 cells and BMVEC (Figure 4). This finding also adds credence to the notion that the immunosuppressive nature of SC may contribute to the ability of ZIKV to establish persistence in the testes.

In summary, this study utilizes LC-MS/MS proteomics as a well-suited complementary approach to define the SC-ZIKV interaction. Our data further demonstrates that the IFN response is critical in mitigating the spread of ZIKV in SC, a finding that may be consequential for therapeutic efforts. Furthermore, MX1 and IFIT1 were identified as marked predictors of the ZIKV-induced antiviral state in SC and as key players in restricting ZIKV replication. Based on our collective data, we propose that the antiviral response in human SC, although slightly delayed and attenuated compared to other human cell types, is sufficient to control ZIKV infection. Therefore, we posit that the apparent ability of ZIKV to persist in the human testes without causing overt inflammation or tissue damage is likely due to both the testicular immune environment, in which both innate and adaptive immune responses are tightly governed, and the ability of ZIKV to antagonize the IFN-I/III pathways in humans.

## Data Availability Statement

The datasets presented in this study can be found in online repositories. The names of the repository/repositories and accession number(s) can be found below: PRIDE database under the identifier PXD025133.

## Author Contributions

DS, HS-A, and SV conceived and designed the study. LC, TK, and MW conducted the proteomics data acquisition and cleaning. DS supervised and conducted the majority of experiments and assays. BJ assisted with experiments and assays. DS wrote the manuscript draft and generated the figures and tables. SV supervised and coordinated the study. DS, BJ, HS-A, LC, TK, MW, and SV edited the manuscript. DS and SV finalized the manuscript for publication. All authors contributed to the article and approved the submitted version.

## Conflict of Interest

The authors declare that the research was conducted in the absence of any commercial or financial relationships that could be construed as a potential conflict of interest.

## Footnotes

1 https://biit.cs.ut.ee/gprofiler/gost

2 https://imagej.nih.gov/ij/
