# Supplementary material for: Paracrine IFN Response Limits ZIKV Infection in Human Sertoli Cells
Source: Front Microbiol. 2021 May 17;12:667146. doi: 10.3389/fmicb.2021.667146 (PMC8165286; doi:10.3389/fmicb.2021.667146)
Supplement: Supplementary file 1 [file Table_1.DOCX]

**Table S1.** Differentially regulated proteins (DRPs) in human Sertoli cells (SC) at 24h post ZIKV infection (related to Figure 1).

| **Accession** | **Gene name** | **Description** | **Abundance Ratio** | **adj. p-value** |
| --- | --- | --- | --- | --- |
| Q96CM8-2 | ACSF2 | Isoform 2 of Acyl-CoA synthetase family member 2, mitochondrial | 2.113 | 2.60E-15 |
| Q15233 | NONO | Non-POU domain-containing octamer-binding protein | 1.666 | 2.52E-06 |

**Table S2.** Differentially regulated proteins (DRPs) in human Sertoli cells (SC) at 72h post ZIKV infection (related to Figure 1).

| **Accession** | **Gene name** | **Description** | **Abundance Ratio** | **adj. p-value** |
| --- | --- | --- | --- | --- |
| P20591 | MX1 | Interferon-induced GTP-binding protein Mx1 | 6.403 | 9.59E-16 |
| P09914 | IFIT1 | interferon-induced protein with tetratricopeptide repeats 1 | 5.457 | 9.59E-16 |
| P05161 | ISG15 | Ubiquitin-like protein ISG15 | 4.653 | 9.59E-16 |
| Q9Y3Z3 | SAMHD1 | deoxynucleoside triphosphate triphosphohydrolase SAMHD1 | 3.849 | 9.59E-16 |
| O95786 | DDX58 | Probable ATP-dependent RNA helicase DDX58 | 2.780 | 9.59E-16 |
| O14879 | IFIT3 | Interferon-induced protein with tetratricopeptide repeats 3 | 2.532 | 9.59E-16 |
| Q01844-5 | EWSR1 | Isoform 5 of RNA-binding protein EWS | 2.325 | 9.59E-16 |
| P42224-1 | STAT1 | Signal transducer and activator of transcription 1-alpha/beta | 2.216 | 9.59E-16 |
| Q8IXQ6 | PARP9 | Poly [ADP-ribose] polymerase 9 | 2.001 | 9.59E-16 |
| O14933 | UBE2L6 | Ubiquitin/ISG15-conjugating enzyme E2 L6 | 1.838 | 6.07E-14 |
| Q460N5 | PARP14 | poly [ADP-ribose] polymerase 14 | 1.821 | 1.16E-13 |
| Q13325 | IFIT5 | Interferon-induced protein with tetratricopeptide repeats 5 | 1.792 | 5.48E-13 |
| P28838 | LAP3 | cytosol aminopeptidase | 1.741 | 1.01E-11 |
| P18465 | HLA-B | HLA class I histocompatibility antigen, B-57 alpha chain | 1.700 | 9.59E-11 |
| P62070-4 | RRAS2 | Isoform 4 of Ras-related protein R-Ras2 | 1.691 | 1.52E-10 |
